# Continuous Compressions, Incomplete Ventilation? A Retrospective Analysis of mCPR in Admitted ED Patients

**DOI:** 10.3390/jcm15030933

**Published:** 2026-01-23

**Authors:** Ingo Voigt, Mehran Babady, Katharina Schütte-Nütgen, Raimund Grondstein, Oliver Bruder

**Affiliations:** 1 Department of Acute and Emergency Medicine, Elisabeth-Hospital Essen, 45138 Essen, Germany; 2 Faculty of Medicine, University of Münster, 48149 Münster, Germany; 3 Department of Cardiology and Angiology, Elisabeth-Hospital Essen, 45138 Essen, Germany; 4 Faculty of Medicine, Ruhr-University Bochum, 44801 Bochum, Germany

**Keywords:** cardiopulmonary resuscitation, out-of-hospital cardiac arrest, resuscitation/methods, ventilation/physiology, emergency medical services

## Abstract

**Background/Objectives**: Mechanical cardiopulmonary resuscitation (mCPR) devices offer consistent chest compressions during prolonged resuscitations and transport, but their impact on ventilation and patient outcomes remains unclear. This study aimed to compare gas exchange, metabolic parameters, and clinical outcomes in patients with ongoing manual versus mechanical CPR upon arrival at the emergency department (ED) after out-of-hospital cardiac arrest (OHCA). **Methods**: We conducted a retrospective analysis of 394 consecutive adult patients with non-traumatic OHCA admitted to a metropolitan cardiac arrest center between January 2019 and December 2024. Patients were categorized into three groups: Return of spontaneous circulation (ROSC) on arrival (n = 240), ongoing manual CPR (n = 107), and ongoing mechanical CPR (n = 47). Gas exchange and metabolic parameters were obtained from initial arterial blood gas (ABG) analysis and monitor readings. The primary outcome was survival to hospital discharge; secondary outcomes included 24 h survival and neurological status at discharge (CPC 1–2). **Results**: Survival to hospital discharge was significantly higher in the manual CPR group (8.4%) compared to 0% in the mechanical CPR group (*p* = 0.04). Both groups showed severe acidosis and hypercapnia upon ED arrival; however, PaCO_2_ levels were significantly higher in the mCPR group (83.0 ± 25.5 mmHg vs. 72.3 ± 21.6 mmHg, *p* = 0.01). ROC analysis identified lactate (AUC = 0.765) and pH (AUC = 0.743) as the strongest predictors of survival, while EtCO_2_ had limited prognostic value (AUC = 0.541). **Conclusions**: In patients with refractory out-of-hospital cardiac arrest admitted with ongoing cardiopulmonary resuscitation, mechanical CPR was associated with higher PaCO_2_ levels on emergency department arrival compared with manual CPR, while other gas exchange parameters did not differ significantly. Given the limited sample size and small number of survivors, these findings are exploratory and hypothesis-generating, underscoring the need for prospective studies on ventilation during continuous chest compressions.

## 1. Introduction

Out-of-hospital cardiac arrest (OHCA) remains a significant public health challenge, with an estimated 100,000 cases occurring annually in Germany and survival rates consistently below 10%. Effective cardiopulmonary resuscitation (CPR) relies on high-quality chest compressions to maintain circulation and adequate ventilation to ensure oxygen delivery and carbon dioxide removal [1]. Refractory out-of-hospital cardiac arrest is characterized by the absence of sustained return of spontaneous circulation (ROSC) despite advanced life support, with ongoing cardiopulmonary resuscitation during transport to the emergency department to facilitate diagnosis of reversible etiologies and the provision of advanced therapeutic options such as pericardiocentesis, thrombolytic therapy for pulmonary embolism, or veno-arterial extracorporeal membrane oxygenation (ECPR). In recent years, resuscitation science and guidelines have placed increasing emphasis on optimizing chest compression quality, focusing on parameters such as rate, depth, and minimization of interruptions [2,3,4].

While manual chest compressions performed by rescuers remain the standard of care, mechanical CPR (mCPR) devices are being used with increasing frequency, particularly in scenarios involving prolonged resuscitation efforts or patient transport. These devices offer the advantage of delivering consistent compressions; however, there is currently no conclusive evidence supporting the overall superiority of mechanical over manual CPR, except in selected subgroups [5,6,7,8].

Although chest compressions are essential, they do not ensure adequate ventilation. Effective ventilation during CPR requires a careful balance. Hyperventilation can raise intrathoracic pressure, reducing venous return and cardiac output, while hypoventilation results in inadequate gas exchange. Both have been shown to be associated with worse outcomes [9,10,11,12]. Current resuscitation guidelines recommend a ventilation rate of approximately 10 breaths per minute with a tidal volume of 500–600 mL when an advanced airway is in place, aiming to avoid both hypo- and hyperventilation [1].

Despite adherence to these recommendations and the use of 100% oxygen, patients often arrive at the emergency department in a state of significant hypoxia and hypercapnia [13,14,15]. This suggests that standard ventilation techniques may be insufficient in the context of refractory cardiac arrest with ongoing chest compressions.

The use of mechanical CPR in combination with mechanical ventilation enables asynchronous ventilation, with ventilatory cycles delivered independently of chest compression phases, and may reduce the risk of operator-dependent hyperventilation compared with manual bag-valve ventilation. However, uninterrupted chest compressions can impair effective lung inflation and carbon dioxide clearance through sustained intrathoracic pressure, intrathoracic airway closure, and reduced dynamic lung compliance, as demonstrated in experimental and translational studies [16,17,18,19].

In a porcine model, continuous chest compressions with asynchronous ventilation were compared to a 30:2 compression-to-ventilation strategy. While, after 30 min of CPR, both groups showed a deteriorating trend in PaO_2_ and rising PaCO_2_ levels, no statistically significant differences between these parameters were observed, suggesting comparable gas exchange under controlled experimental conditions [20]. However, whether these findings translate to real-world human resuscitations remains uncertain.

We hypothesized that the method of CPR (manual vs. mechanical) during transport could lead to measurable differences in blood gases and acid-base balance upon ED arrival, and that these differences might be associated with patient outcomes.

## 2. Materials and Methods

We analyzed data from consecutive out-of-hospital cardiac arrest (OHCA) cases admitted to the emergency department of a metropolitan German cardiac arrest center between January 2019 and December 2024. Data were extracted from the local resuscitation registry, which is based on the German Resuscitation Registry. The study was approved by the Ethics Committee of the Medical Association North Rhine (Nr. 54/2025), with a waiver of informed consent due to its retrospective design.

**Figure 1:**
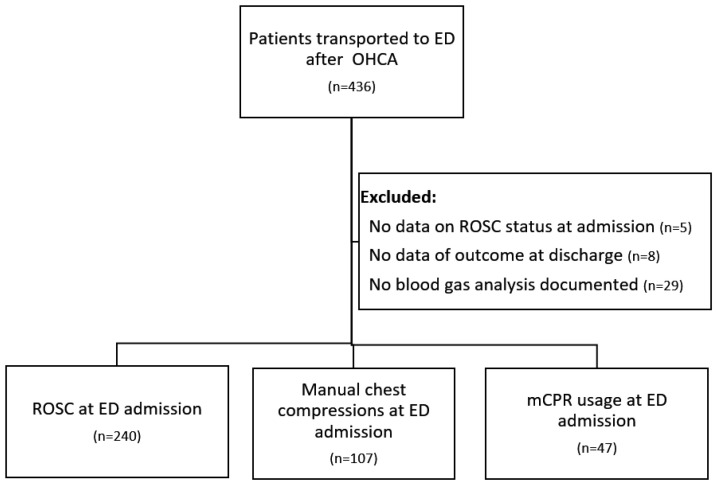
Patient inclusion flowchart.

We included adult patients (age > 18 years) with non-traumatic out-of-hospital cardiac arrest who were transported to the ED either with sustained ROSC or with ongoing CPR. Patients with unknown status of ROSC at ED admission or unknown survival outcome at discharge were excluded. Patients meeting criteria were categorized into three groups for further analysis (Figure 1):

**Group 1—ROSC on Arrival**: Patients who had achieved return of spontaneous circulation by the time of ED admission.

**Group 2—Ongoing CPR (Manual)**: Patients who arrived with ongoing resuscitation using manual chest compressions by emergency medicine service (EMS).

**Group 3—Ongoing CPR (Mechanical)**: Patients who arrived with ongoing resuscitation using a mechanical CPR device (mCPR). Mechanical CPR was performed using piston-driven mechanical chest compression devices, including the LUCAS^®^ system (Stryker, Kalamazoo, MI, USA) and the corpuls^®^ CPR device (GS Elektromedizinische Geräte G. Stemple GmbH, Kaufering, Germany), delivering continuous chest compressions in accordance with current resuscitation guidelines.

Using the registry and electronic patient records of the hospital information system, we extracted data on patient characteristics and resuscitation details. Neither consistently recorded ventilator settings nor a sufficient number of ventilator parameter datasets from the prehospital setting and the emergency department were available; consequently, analyses address gas exchange and acid–base status rather than the applied ventilation strategy.

Blood gas–derived measures of gas exchange and acid–base status at ED arrival were obtained from the initial arterial blood gas (ABG) analysis, complemented by non-invasive monitor readings. These included: end-tidal carbon dioxide (EtCO_2_), oxygen saturation (SpO_2_, %), arterial pH, partial pressure of carbon dioxide (PaCO_2_, mmHg), and lactate (mmol/L). In cases where extreme ABG values exceeded device measurement ranges (e.g., PaCO_2_ > 125 mmHg or pH < 6.9), those data were excluded from analysis. Exact low-flow time prior to ABG sampling was unavailable due to retrospective registry design. Time from emergency call to ED admission was used as a surrogate parameter. Therapeutic measures in the acute post resuscitation phase were documented. Cerebral Performance Category (CPC) score at discharge was recorded for survivors, with CPC 1–2 considered a favorable neurologic outcome.

The primary outcome was survival to hospital discharge (alive, regardless of neurologic status). Secondary outcomes included 24 h survival and favorable neurological outcome at discharge (CPC 1–2). However, our comparisons of interest were between Group 2 and Group 3, in terms of their ventilation parameters and outcomes.

We summarized continuous variables as mean ± standard deviation (SD) or median with interquartile range (IQR) if non-normal distribution and categorical variables as counts and percentages. For comparing the manual vs. mechanical CPR groups, we used Student’s t-test for normally distributed continuous data, or Mann–Whitney U test if distributions were non-normal. Categorical variables were compared with chi-square or Fisher’s exact tests, as appropriate. A two-tailed *p*-value < 0.05 was considered statistically significant. Although three groups were identified, our primary analysis directly compared the two ongoing CPR groups (Group 2 vs. Group 3) to address the study question; we report *p*-values for these comparisons. Descriptive data for the ROSC group (Group 1) are provided for context but were not included in the significance testing for ventilation parameters or outcomes between CPR methods.

All analyses were performed using MedCalc^®^ Statistical Software version 23.1.3 (MedCalc Software Ltd., Ostend, Belgium; 2025).

## 3. Results

### 3.1. Patient Characteristics

During the study period, data from 436 consecutive out-of-hospital cardiac arrest (OHCA) cases were documented in the local resuscitation registry. A total of 394 patients met the inclusion criteria. Of these, 240 patients (60.1%) achieved return of spontaneous circulation (ROSC) before or upon arrival at the emergency department (Group 1), 107 patients (27.2%) arrived with ongoing manual CPR (Group 2), and 47 patients (11.9%) with ongoing mechanical CPR (Group 3).

Baseline characteristics were comparable between the manual and mechanical CPR groups (Table 1). The mean age was 63.7 ± 16.6 years in the manual group and 65.5 ± 15.8 years in the mechanical group (*p* = 0.53), with a predominance of male patients in both groups (71.9% vs. 78.7%, *p* = 0.37). The proportion of witnessed arrests did not differ significantly (60.7% manual vs. 59.6% mechanical, *p* = 0.89), and initial shockable rhythms were observed in approximately one-third of cases in both groups (33.6% manual vs. 36% mechanical, *p* = 0.77).

The mean time from emergency call to arrival at the emergency department was comparable between the manual CPR group (51.7 ± 17 min) and the mechanical CPR group (53.8 ± 13 min), with no statistically significant difference (*p* = 0.66).

### 3.2. Resuscitation Interventions and ED Course

Both ongoing CPR groups were treated with similar doses of epinephrine (6.1 ± 4.3 mg for manual CPR, 6.9 ± 3.8 mg for mCPR, *p* = 0.27) and amiodarone (334 ± 142 mg vs. 342 ± 77 mg, *p* = 0.71) as well as counts of shocks applied for shockable rhythms (*p* = 0.68). On ED arrival, only 12.2% of manual CPR and 8.5% of mechanical CPR patients still had a shockable rhythm (*p* = 0.51). Among patients without ROSC at ED arrival, ECPR using VA-ECMO was applied at similar rates in the manual and mechanical CPR groups (18.7% vs. 17%; *p* = 0.80). VA-ECMO was additionally used in a small proportion of patients with ROSC at admission (3.8%), mainly for profound cardiogenic shock. With a comparable proportion of STEMI in both groups (20.6% vs. 21.2%, *p* = 0.93) coronary angiography was performed in 25.2% of manual CPR and 25.5% of mechanical CPR patients (*p* = 0.96), with PCI in 16.6% vs. 19.1%, respectively (*p* = 0.71). Targeted temperature management (TTM) was initiated at similar rates (19.6% vs. 21.3%, *p* = 0.81). Thrombolytic therapy for suspected pulmonary embolism was rare (8.4% in manual CPR vs. 0% in mCPR, *p* = 0.13).

### 3.3. Gas Exchange and Metabolic Parameters on ED Arrival

The end-tidal CO_2_ (EtCO_2_), reflecting pulmonary blood flow and CO_2_ elimination, was slightly higher in the mechanical CPR group compared to the manual CPR group (Table 2). Oxygen saturation (SpO_2_) upon arrival tended to be lower in the mechanical CPR group (77.4 ± 17.2%) versus the manual group (80.1 ± 14.6%), missing statistical significance (*p* = 0.31).

Arterial blood gas measurements revealed severe acidosis and hypercapnia in both groups. The average pH showed no statistical significance in both groups (6.94 ± 0.13 manual group vs. 6.91 ± 0.17 mCPR; *p* = 0.23). The corresponding PaCO_2_ levels were elevated in both groups with a statistically significant higher hypercapnia in the mCPR group (72.3 ± 21.6 mmHg vs. 83.0 ± 25.5 mmHg; *p* = 0.01).

Lactate levels on arrival were extremely high in both cohorts, reflective of prolonged low-flow state and anaerobic metabolism. The manual CPR group had a mean lactate of 12.7 ± 3.9 mmol/L, and the mechanical CPR group a mean of 11.7 ± 4.8 mmol/L (*p* = 0.17).

### 3.4. Survival Outcomes

Survival outcomes for manual vs. mechanical CPR are shown in Table 3. There was no significant difference in short-term survival between the manual CPR group (n = 28;26.1%) and the mCPR group (n = 8;17%) (*p* = 0.22), respectively. Survival to hospital discharge was significantly higher in the manual CPR group (8.4%) compared to the mCPR group (0%) (*p* = 0.04).

**Figure 2:**
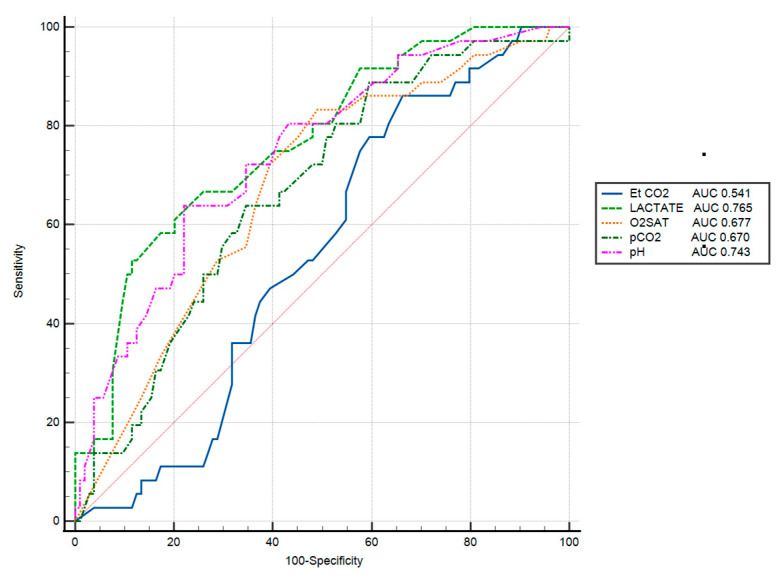
ROC curves of gas exchange and metabolic parameters on survival.

Receiver operating characteristic (ROC) curve analysis was performed to evaluate the predictive performance of various ventilation and metabolic parameters for outcome discrimination. The area under the curve (AUC) was highest for lactate (AUC = 0.765), followed by pH (AUC = 0.743), oxygen saturation (O2SAT) (AUC = 0.677), and PaCO_2_ (AUC = 0.670). In contrast, end-tidal CO_2_ (EtCO_2_) showed poor predictive value, with an AUC of 0.541 (Figure 2).

## 4. Discussion

In this retrospective study of patients with refractory out-of-hospital cardiac arrest (OHCA) transported to the emergency department, we compared clinical outcomes and ventilation parameters between those receiving manual chest compressions and those transported with ongoing mechanical CPR (mCPR). Our analysis revealed two key findings.

First, overall outcomes differed significantly between the groups. While short-term survival was similar, none of the patients in the mCPR group survived to hospital discharge, compared to a 7% survival rate in the manual CPR group.

Second, patients treated with mCPR showed significantly more extreme values of ventilation-associated parameters. This was reflected by higher PaCO_2_ levels, as well as trends toward more pronounced respiratory acidosis and hypoxemia, despite the use of advanced airway management and administration of 100% oxygen. ROC-Analysis revealed the potential negative effect of PaCO_2_ levels on survival.

These findings suggest that mechanical CPR may be associated with less effective ventilation during prolonged resuscitation efforts, potentially contributing to poorer outcomes.

Persistent hypercapnia and acidosis during prolonged resuscitation as well as their negative impact on survival have been shown in multiple studies [11,12,21]. Elevated PaCO_2_ may lead to cerebral vasodilation, increased intracranial pressure, and exacerbation of cerebral edema, while hypoxemia may result in systemic tissue injury. Post-arrest studies have demonstrated a U-shaped relationship between PaCO_2_ and outcome, emphasizing the importance of avoiding both hyper- and hypocapnia [22,23]. Normocapnia is generally regarded as the optimal target to minimize secondary brain injury [1].

Several physiological mechanisms may underline the observed differences in gas exchange between manual and mechanical CPR. First, manual CPR may result in brief interruptions during the change in the person performing the cardiac massage or in reduced depth of chest compression due to muscular fatigue, enabling chest recoil and transient reductions in intrathoracic pressure. Both mechanisms may support more effective ventilation. In contrast, mCPR delivers continuous, uniform compressions that maintain elevated intrathoracic pressure, potentially impairing alveolar ventilation and gas exchange [5,6,7]. Second, animal studies have demonstrated progressive reductions in PaO_2_ and EtCO_2_ during ongoing compressions, attributed to dynamic atelectasis, progressive reduction in functional lung volume, and ventilation-perfusion mismatch [17,18,20]. Third, intrathoracic airway closure during continuous compressions may further inhibit effective ventilation [16,24]. Magliocca et al. reported that mechanical CPR induced significant acidosis, hypercapnia, and hypoxemia in a porcine model, with a marked decline in respiratory system compliance despite unchanged chest wall compliance. Furthermore, during mCPR, pulmonary shunt fraction rose by up to 30%, indicating redistribution of perfusion toward poorly or non-ventilated lung units. Histopathological and radiological findings confirmed the presence of CPR-associated lung edema (CRALE) [18]. CRALE is increasingly recognized as a complication of prolonged resuscitation, with clinical features including bilateral infiltrates on imaging, PaO_2_/FiO_2_ ratios < 300 mmHg, reduced lung compliance, and elevated dead space [25,26,27]. These changes mirror those seen in ICU patients with post-arrest lung injury and suggest a direct contribution to impaired ventilation.

Current guidelines recommend a ventilation rate of 10 breaths per minute with tidal volumes of approximately 500 mL during CPR. Ventilation is typically delivered via bag-valve-mask (BVM) or bag-attached advanced airway devices [1,4]. However, manual ventilation is highly operator-dependent and frequently results in inconsistent tidal volumes and rates, even among trained providers [13,28].

Conventional ventilators are not optimized for use during CPR. In volume-controlled modes, chest compressions may trigger premature cycling or incomplete tidal delivery due to pressure limitations. In pressure-controlled modes, external forces can result in overshooting airway pressures or large variability in tidal volume [2,13,19,29]. A six-point strategy has been proposed to optimize ventilation during CPR, focusing on settings such as PEEP, tidal volume, FiO_2_, respiratory rate, peak inspiratory pressure, and inspiratory time [30]. Specialized modes such as continuous chest compression synchronized ventilation (CCSV) have shown promise in animal studies, but human data remain limited [29,31,32,33].

The application of PEEP during CPR remains controversial. While not currently addressed in guidelines, moderate PEEP may prevent alveolar collapse, increase functional residual capacity, and improve oxygenation during low-flow states. A 2022 porcine study comparing PEEP levels of 0, 8, and 16 mbar during mCPR found that higher PEEP improved lung ventilation, lowered driving pressures, and reduced lung injury without compromising mean arterial pressure [34]. However, this benefit was offset by increased PaCO_2_ levels, raising concerns about ventilation adequacy.

In our cohort, both groups presented with severe hypercapnia, acidosis, and hypoxemia upon ED arrival. Slightly higher EtCO_2_ values in the mCPR group could indicate either better chest perfusion or on the other hand CO_2_ trapping due to inadequate ventilation. Similarly, higher PaCO_2_ in mCPR patients likely reflects impaired gas exchange caused by elevated intrathoracic pressure and reduced dynamic lung compliance during uninterrupted compressions.

Transporting patients with refractory cardiac arrest under continuous chest compressions is often necessary when out-of-hospital extracorporeal VA-ECMO cannulation is not feasible, to enable in-hospital initiation of ECPR. However, this approach may lead to prolonged low-flow times, increased logistical complexity during transport, and potential physiological disadvantages due to ongoing compressions without effective ventilation.

Despite a similar rate of VA-ECMO usage across both groups in our study, survival to hospital discharge remained poor. This highlights the critical importance of both patient selection and timely intervention. Current recommendations suggest that the interval from cardiac arrest to establishment of full-flow VA-ECMO should be less than 60 min [35,36]. However, this threshold is frequently exceeded, as demonstrated in recent clinical trials [37,38,39]. Notably, a pre-cannulation PaCO_2_ > 70 mmHg has been associated with poorer outcomes, emphasizing the need for early intervention and optimal ventilation management during transport [40].

### Limitations

This study has several limitations due to its retrospective design. First, it was a single-center analysis with a relatively small sample of patients in the mechanical CPR group, which limits statistical power and generalizability. The use of mechanical CPR was not randomized but rather determined by EMS crews based on availability and clinical judgment, introducing potential selection bias. We attempted to account for arrest severity by considering initial rhythm and total epinephrine dose, but other unmeasured confounders (e.g., quality of manual CPR, operator skill, or specific CPR protocols) could have influenced outcomes. Although baseline characteristics were statistically comparable, subtle differences in reversible etiologies cannot be excluded and may have contributed to the observed results. Data on prehospital ventilation parameters such as exact ventilation rate, tidal volume, or airway management technique were not available, so we cannot directly compare the quality of ventilation delivered in the field between groups. We also lacked information on certain clinical factors that might affect ventilation during CPR, such as the presence of severe pulmonary comorbidities, obesity or the occurrence of CPR-related injuries like pneumothorax or airway obstruction. We included only validated ABG data and excluded extreme outlier values thought to be due to measurement limitations; while this improves data reliability, it might discard some true extreme physiology. Additionally, our outcome of neurological status was only assessed at hospital discharge; longer-term neurological follow-up was not within the scope of this study. Despite these limitations, our study provides novel data by focusing on ventilation and gas exchange parameters in a real-world cohort of ongoing CPR patients, a group that is underrepresented in resuscitation literature.

## 5. Conclusions

In this retrospective single-center analysis of patients with refractory out-of-hospital cardiac arrest admitted to the emergency department with ongoing cardiopulmonary resuscitation, mechanical CPR during transport was associated with higher PaCO_2_ levels on ED arrival compared with manual CPR, while other gas exchange and acid–base parameters did not differ significantly.

Although survival to hospital discharge was observed only in the manual CPR group, the limited sample size and small number of survivors preclude definitive outcome comparisons. Accordingly, these findings should be interpreted as exploratory and hypothesis-generating, rather than indicative of causal effects or device superiority.

The observed association between mechanical CPR and impaired carbon dioxide clearance highlights the need for further prospective studies focusing on ventilation strategies during continuous chest compressions, particularly during prolonged transport and in patients considered for advanced rescue therapies such as extracorporeal CPR. Optimizing ventilation under real-world resuscitation conditions may represent an important target to improve physiological stability and outcomes in refractory cardiac arrest.

## Figures and Tables

**Table 1:** Preclinical data of Out-Of-Hospital-Cardiac-Arrest patients transported to the emergency department.

	ROSC	Manual Compression	mCPR	*p*-Value
Patients, n (%)	240 (60.9)	107 (27.2)	47 (11.9)	
Male, n (%)	164 (68.3)	77 (72.0)	37 (78.7)	0.37
Age (years, mean ± SD)	68.1 ± 14.6	63.7 ± 16.6	65.5 ± 15.8	0.53
Witnessed cardiac arrest, n (%)	168 (70)	65 (60.7)	28 (59.6)	0.89
Initial rhythm shockable (VF/VT), n (%)	81 (33.8)	36 (33.6)	17 (36)	0.77
Adrenaline (mg, mean ± SD)	3.1 ± 2.1	6.1 ± 4.3	6.9 ± 3.8	0.27
Amiodarone (mg, mean ± SD)	330 ± 71	334 ± 142	342 ± 77	0.71
Shocks given (N ± SD)	1.9 ± 0.8	2.7 ± 1.5	2.6 ± 1.1	0.68
Time from call to hospital admission (minutes ± SD)	58 ± 20	51 ± 17	53 ± 13	0.66
Shockable rhythm (VF/VT) at ED admission, n (%)	1 (0.4)	13 (12.1)	4 (8.5)	0.51
eCPR /ECLS, n (%)	9 (3.8)	20 (18.7)	8 (17.0)	0.80
TTM, n (%)	106 (41.6)	21 (19.6)	10 (21.3)	0.80
Lysis for pulmonary embolism, n (%)	8 (3.3)	9 (8.4)	0 (0)	0.12
STEMI, n (%)	65 (27.0)	22 (20.6)	10 (21.2)	0.93
Coronary angiography, n (%)	102 (42.5)	27 (25.2)	12 (25.5)	0.96
PCI, n (%)	74 (30.8)	18 (16.8)	9 (19.1)	0.70

ROSC return of spontaneous circulation, VF/VT ventricular fibrillation/ventricular tachycardia, STEMI ST-elevation myocardial infarction, TTM targeted temperature management, ECLS extracorporeal life support, PCI percutaneous coronary intervention).

**Table 2:** Gas exchange and metabolic values at ED admission.

	ROSC	Manual Compression	mCPR Device Use	*p*-Value
EtCO_2_ (mmHg, median [IQR])	38 [36.5–42.4]	25 [19.9–35.0]	30 [17.4–37.5]	0.84
Sp0_2_ (%, mean ± SD)	93.5 ± 8.3	80.1 ± 14.6	77.4 ± 17.2	0.31
pH (mean ± SD)	7.12 ± 0.21	6.94 ± 0.13	6.91 ± 0.17	0.23
pCO_2_ (mmHg, mean ± SD)	62.5 ± 22.7	72.2 ± 21.7	83.0 ± 25.5	0.01
Lactate (mmol/L, mean ± SD)	8.3 ± 4.2	12.7 ± 3.9	11.7 ± 4.8	0.17
BE (mmol/L, median [IQR])	−9 [−11; −9]	−13 [−18; −15]	−13 [−18; −6]	0.20

**Table 3:** Comparison of outcome parameter.

	ROSC	Manual Compression	mCPR Device Use	*p*-Value
24 h survival, n (%)	194 (80.8)	28 (26.1)	8 (17.0)	0.22
Discharged alive, n (%)	86 (35.8)	9 (8.4)	0 (0)	0.04
CPC 1/2, n (%)	71 (29.5)	8 (7.5)	0 (0)	0.05
Withdrawal of therapy, n (%)	135 (56.3)	33 (30.8)	14 (29.8)	0.91

CPC Cerebral Performance Category.

## Data Availability

The raw data supporting the conclusions of this article will be made available by the authors on request.

## Abbreviations

The following abbreviations are used in this manuscript:

mCPR	Mechanical cardiopulmonary resuscitation
ED	Emergency Department
OHCA	Out-of-hospital cardiac arrest
ROSC	Return of spontaneous circulation
TTM	Targeted temperature management
BVM	Bag-valve-mask
PEEP	Positive End-Expiratory Pressure
CCSV	Chest compression synchronized ventilation
eCPR	Extracorporeal cardiopulmonary resuscitation
EMS	Emergency medical service
